# Analysis of T-DNA alleles of flavonoid biosynthesis genes in *Arabidopsis* ecotype Columbia

**DOI:** 10.1186/1756-0500-5-485

**Published:** 2012-09-04

**Authors:** Peter A Bowerman, Melissa V Ramirez, Michelle B Price, Richard F Helm, Brenda SJ Winkel

**Affiliations:** 1 Department of Biological Sciences, Blacksburg, VA, 24061, USA; 2 Department of Biochemistry, Virginia Tech, Blacksburg, VA, 24061, USA; 3 Current address: Department of Biology, Colorado State University, Fort Collins, CO, 80523, USA; 4 Current address: Department of Microbiology, Immunology & Pathology, Colorado State University, Fort Collins, CO, 80523, USA; 5 Current address: Department of Plant Pathology, Physiology, and Weed Science, Virginia Tech, Blacksburg, VA, 24061, USA

**Keywords:** Arabidopsis, Ecotype, Insertional inactivation lines, Flavonoid, Transparent testa

## Abstract

**Background:**

The flavonoid pathway is a long-standing and important tool for plant genetics, biochemistry, and molecular biology. Numerous flavonoid mutants have been identified in *Arabidopsis* over the past several decades in a variety of ecotypes. Here we present an analysis of *Arabidopsis* lines of ecotype Columbia carrying T-DNA insertions in genes encoding enzymes of the central flavonoid pathway. We also provide a comprehensive summary of various mutant alleles for these structural genes that have been described in the literature to date in a wide variety of ecotypes.

**Findings:**

The confirmed knockout lines present easily-scorable phenotypes due to altered pigmentation of the seed coat (or testa). Knockouts for seven alleles for six flavonoid biosynthetic genes were confirmed by PCR and characterized by UPLC for altered flavonol content.

**Conclusion:**

Seven mutant lines for six genes of the central flavonoid pathway were characterized in ecotype, Columbia. These lines represent a useful resource for integrating biochemical and physiological studies with genomic, transcriptomic, and proteomic data, much of which has been, and continues to be, generated in the Columbia background.

## Background

Flavonoids are a group of specialized plant metabolites that play critical roles in plant reproduction, defense from abiotic and biotic stress and are of growing interest as health-promoting compounds in human and animal diets [1-3]. As pigments, they have also figured into numerous seminal biological discoveries including Mendel’s elucidation of the laws of genetics, McClintock’s discovery of mobile genetic elements, and more recently the phenomenon of cosuppression, or RNA interference, in *Petunia hybrida* (reviewed in [4,5]). The flavonoid pathway continues to serve as an important experimental system in a variety of plant species, with studies ranging from understanding complex transcriptional control to biochemical structure-function relationships, intra- and intercellular transport, and the subcellular organization of pathways as multi-enzyme complexes [6-9]. Still, many questions remain about the specific biological targets of flavonoids in plants and animals [1,10], while engineering the production of specific flavonoids in plants and microorganisms is still far from straight-forward [11,12].

**Figure 1:**
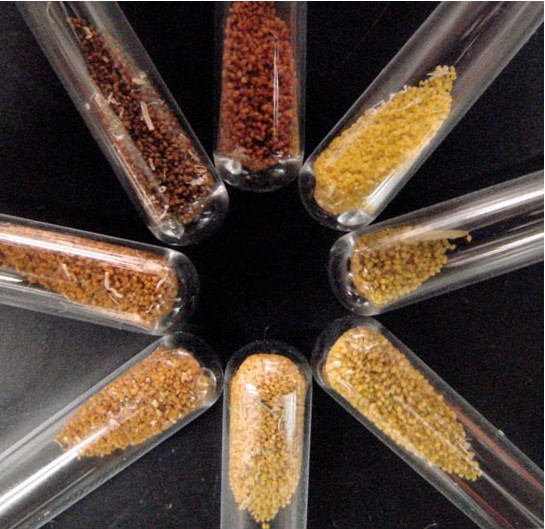
**Seed coat color phenotype of confirmed homozygous T-DNA lines with insertions disrupting genes involved in flavonoid biosynthesis.** From top center, clockwise seeds are: Col-0 WT, *tt4-13*, *tt5-3*, *tt5-2*, *tt6-3*, *tt7-5*, *tt11-11*, and *ban-4*.

Mutations within genes in the flavonoid biosynthetic pathway of *Arabidopsis* were described as early as 1971, easily identified by the *transparent testa* (*tt*) phenotype of the mutant seed coat [13] (Figure 1 and Table 1). Large-scale mutant screens carried out by Maarten Koornneef, initially aimed at characterizing the effects of fast-neutron and X-rays, identified many more flavonoid biosynthetic and regulatory genes [14,15]. Several other mutants were subsequently identified by Koornneef and others, almost all which have now been cloned and characterized [2]. While this represented an extremely useful toolset, these EMS and fast neutron induced mutations were isolated in a variety of ecotypes, primarily Landsberg but also several others, complicating the analysis of differences between mutants. While differences between ecotypes are sometimes minimal, morphological differences between ecotypes can be easily identified by eye, and research indicates that there are important differences between these backgrounds [16-18]. Here we describe the confirmation and preliminary characterization of mutant alleles for genes encoding flavonoid enzymes in *Arabidopsis* ecotype Columbia-0 (Col-0) that are available as part of the SALK collection of T-DNA insertion lines [19]. These lines represent a useful set of tools for analyzing the organization of flavonoid biosynthetic enzymes and their end products, as well the cellular, physiological and ecological roles of flavonoids. We also present a compilation of mutant alleles for flavonoid structural gene that have been described in the literature to date in a variety of different ecotypes.

**Table 1:** **Summary of enzyme-encoding *tt *alleles described to date**

**Gene**	**Allele**^**1**^	**Line number**^**2**^	**Ecotype**^**3**^	**Mutagen**^**4**^	**First described**
chalcone synthase (CHS)at5g13930	*tt4-1*	85	Ler	EMS	[14,20]
	*tt4-2*	2YY6	Col	EMS	[21-23]
	*tt4-3*	C1	Col	Carbon ions	[24]
	*tt4-4*	C2	Col	Carbon ions	
	*tt4-5*	UV01	Ler	γ radiation	[25]
	*tt4-6*	UV25	Ler	EMS	
	*tt4-7*	UV113	Ler	γ radiation	
	*tt4-8*	UV118a	Ler	γ radiation	
	*tt4-9*	38G1R	Ler	γ radiation	
	*tt4-10*		Est-1	EMS	[26]
	*tt4-11*	DFW34	Ws-2	T-DNA	[27]
	*tt4-12*	CS429127 / GK-304D03	Col	T-DNA	[28]
	***tt4-13***	**SALK_020583**^**5**^	**Col-0**	**T-DNA**	[29,30]
	*tt4-14* through *21*			zinc finger nucleases	[31]
chalcone isomerase (CHI)at3g55120	*tt5-1*	86		EMS	[14]
	***tt5-2***	**CS300857/ GK-176H03**	**Col**	**T-DNA**	[28,30]**; this report**
	***tt5-3***	**SALK_034145**	**Col-0**	**T-DNA**	**This report**
flavanone 3-hydroxylase (F3H)at3g51240	*tt6-1*	87	Ler	EMS	[14,32]
	f3h-2::En		Col	Transposon	[32]
	f3h-3::En		Col	Transposon	
	f3h-4f		Col	Transposon	
	f3h-5f		Col	Transposon	
	*tt6-2*	CS427992 / GK-292E08	Col-0	T-DNA	[28]
	***tt6-3***	**SALK_113904**^**5**^	**Col-0**	**T-DNA**	[33]
	*tt6-4*	SALK_023664	Col-0	T-DNA	Leaky allele – unpublished results
flavonoid 3'-hydroxylase (F3'H)at5g07990	*tt7-1*	88	Ler	EMS	[14,34]
	*tt7-2*		Col-7	T-DNA	[35]
	*tt7-3*	CS433473 / GK-349F05	Col-0	T-DNA	[28,30]
	*tt7-4*	DJI11	Ws-2	T-DNA	[27]
	***tt7-5***	**SALK_053394**	**Col-0**	**T-DNA**	[36]
dihydroflavonol 4-reductase (DFR) at5g42800	*tt3-1*	84	Ler	EMS	[37]
	*tt3-2*	CS428258 / GK-295C10	Col-0	T-DNA	[28]
	*tt3-3*		Est-1	fast neutrons	[26]
		GK-212G01	Col-0	T-DNA	Some segregants have pale brown seeds, none yellow
		SALK_099848	Col-0	T-DNA	Does not have phenotype
anthocyanidin synthase (ANS/LDOX)at4g22880	*tt11-1*				Debeaujon and Koornneef, unpublished
	*tt11-2*		Ler	EMS	[38]
	*tds4-1*		Ws-4	T-DNA but not tagged (INRA)	[35]
	*tds4-2*	SALK_028793	Col-0	T-DNA	[39]
	*tds4-3*	CSHL GT9767	Ler	Gene trap	
	*tt17*		Est-1	Fast neutrons	[26]
	*tt18-1*	AB084467	Col	Carbon ions	[40]
	*tt18-2*	AB084468	Col	Carbon ions	
	*tt18-3*		Col	Carbon ions	
	***tt11-11 (tds4-4)***	**SALK_073183**	**Col-0**	**T-DNA**	[39]**; this report.**
anthocyanidin reductase (ANR/BAN) at1g61720	*ban-1*		Ws-2	T-DNA	[41]
	*ban-2*	F36	En-1	unknown	[42]
	*ban-3*	F52	En-1	unknown	
	***ban-4***	**SALK_040250**^**5**^	**Col-0**	**T-DNA**	**This report**
flavonol synthase 1 (FLS1)at5g08640	*fls1-1*	fls-1::En	Col	Transposon	[32,43]
	*fls-2f*		Col	Transposon	[32]
	*fls-3f*		Col	Transposon	
	*fls-4d*		Col	Transposon	
	*fls1-2*	RIKEN PST16145	No-0	T-DNA	[44]
	***fls1-3***	**INRA FLAG_533E06 (AJ588535/EGT283)**	**Ws**	**T-DNA**	[45]
		SALK_076420	Col-0	T-DNA	Recessive embryo lethal potentially due to disruption of adjacent divergently-transcribed gene [45]
FLS2at5g63580	*fls2-1*	SALK_023235	Col-0	T-DNA	[45]
	*fls2-2*	GK-429B10	Col-0	T-DNA	[44]
FLS3at5g63590	*fls3-1*	SALK_050041	Col-0	T-DNA	[44,45]
FLS4at5g63595	*fls4-1*	SALK_002309	Col-0	T-DNA	
FLS5at5g63600	*fls5-1*	CS430396 / GK-317E12	Col-0	T-DNA	
FLS6at5g43935	*fls6-1*	SALK_003879^5^	Col-0	T-DNA	

^1^ Alleles in bold are described in the current study.

^2^ GK = GABI-Kat.

^3^Standard ecotype abbreviations, as follows: Landsberg erecta (Ler); Columbia accession number 0 (Col-0) or accession unknown (Col), ; Enkheim (En-1); Estland (Est-1); Wassilewskija (Ws-2).

^4^ ethyl methanesulfonate (EMS).

^5^ independently-derived homozygote already available at ABRC.

## Findings

### Confirmation of homozygous *tt* alleles

T-DNA insertion lines in ecotype Col-0 were obtained from the Arabidopsis Biological Resource Center (ABRC, Columbus, OH) for genes encoding six of the eight enzymes of the central flavonoid pathway: chalcone synthase (CHS, SALK_020583), chalcone isomerase (CHI, SALK_034145 and CS300857 from the GABI-Kat project), flavanone 3-hydroxylase (F3H, SALK_113904), flavonoid 3^′^-hydroxylase (F3^′^H, SALK_053394), anthocyanidin synthase (ANS, SALK_073183), and anthocyanidin reductase (ANR, SALK_040250). These lines were assigned allele numbers based on the previously-published alleles for each locus (Table 1). Note that a mutant allele for dihydroflavonol reductase (DFR) was recently identified in the Col-0 background that was not included in this study; no stable mutant allele has yet been identified in this ecotype for flavonol synthase 1 (FLS1).

**Figure 2:**
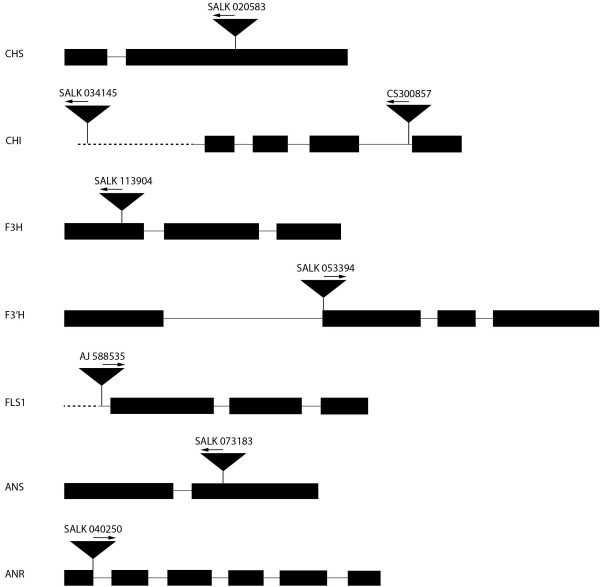
**Schematic of homozygous T-DNA insertion lines.** Boxes indicate exons, solid lines indicate introns and 5^′^ leader sequence, and dashed lines indicate genomic sequence. Insertion sites are indicated by black triangles. The arrows above the insertion indicate the direction of the T-DNA left-border primer sequence used for mapping the insertion sites. The fls1 line is described in Owens et al. [45]. Genes are chalcone synthase (*CHS*), chalcone isomerase (*CHI*), flavanone 3 hydroxylase (*F3H*), flavonoid 3^′^ hydroxylase (*F3′H*), flavonol synthase (*FLS1*), anthocyanidin synthase (*ANS*), and anthocyanidin reductase (*ANR*).

**Figure 3:**
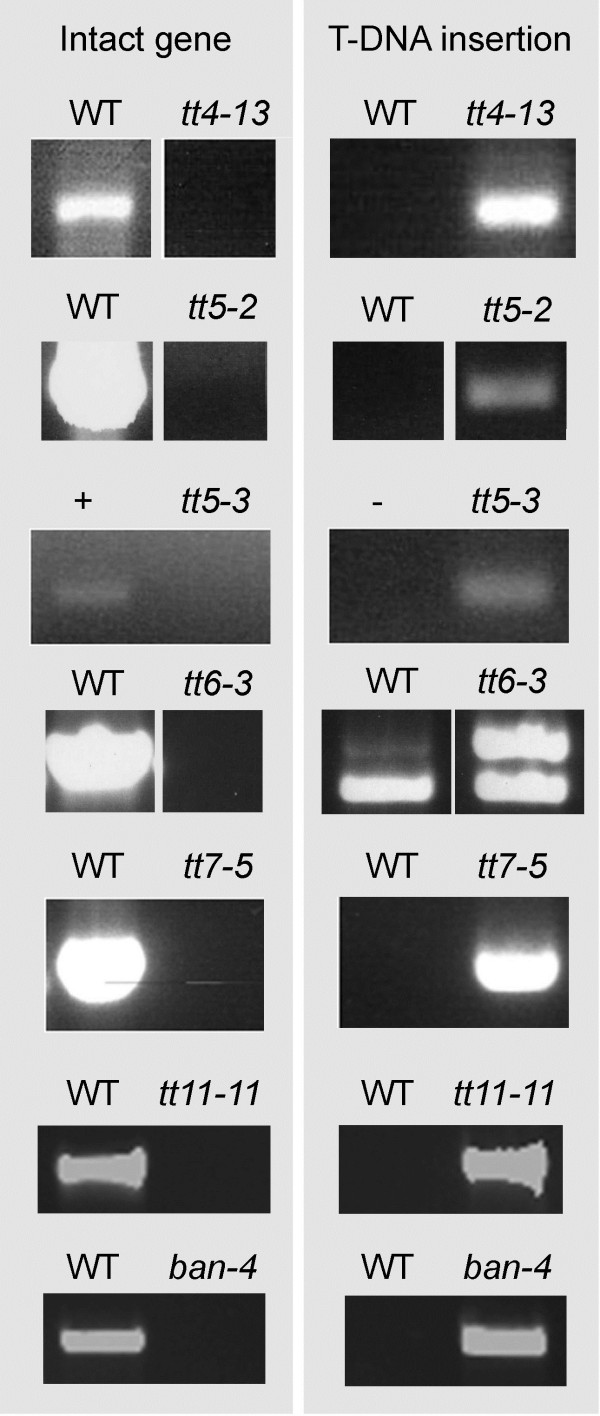
**PCR confirmation of homozygous insertion lines for described *tt *alleles.** T-DNA insertions were confirmed using T-DNA and gene-specific primers, while intact genes were confirmed using two gene-specific primers spanning the mapped T-DNA insertion site. Homozygous lines are indicated by the presence of a T-DNA insertion but not an intact gene.

DNA was isolated from leaves of each T-DNA line to screen for lines homozygous for each insertion. The ability to produce a PCR product from Col-0 wild-type plants using primers that span the T-DNA insertion site (Figure 2) was used to identify the presence of an intact gene. The absence of an amplicon using the same primers for T-DNA lines indicates that the insertion is present, while products generated using one T-DNA-specific and one gene-specific primer indicate the presence of a T-DNA insertion in the gene of interest. The results illustrated in Figure 3 identify each line as containing a homozygous T-DNA insertion in the gene of interest, most within the respective open reading frames, with the exception of alleles of *CHI* (SALK_034145) and *FLSI* (AJ588535)*,* which contain insertions within the promoters, and *CHI* (CS300857) and *ANR* (SALK_040250) with insertion in introns. It should be noted that these lines may contain additional T-DNA insertions at other sites of the genome; it has not yet been determined whether that is the case for any of the lines described here.

### End product and pigmentation analyses of *tt* alleles

**Figure 4:**
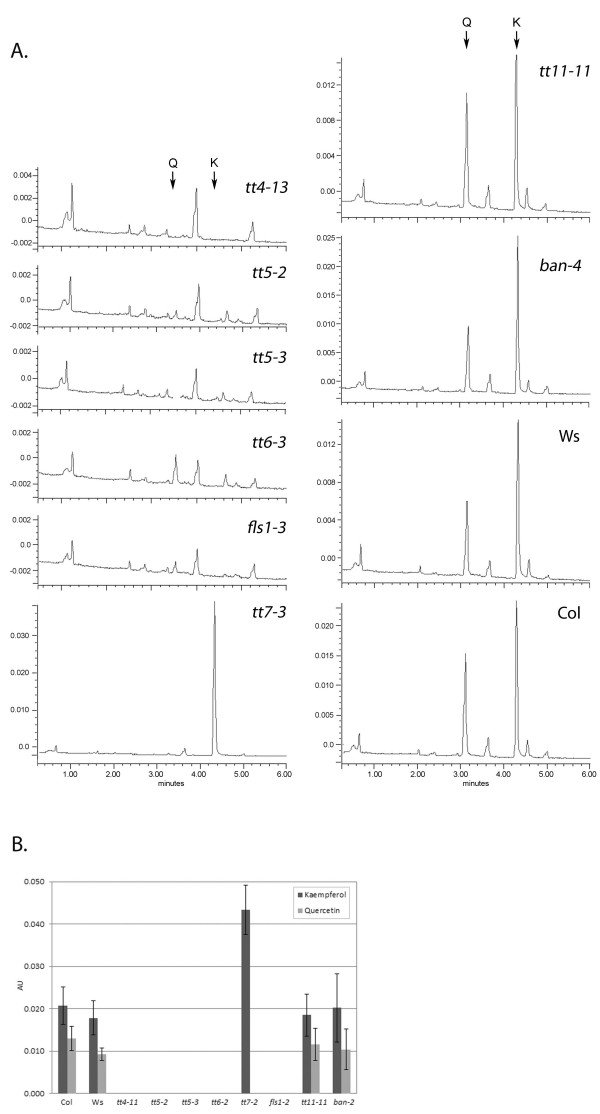
**UPLC analysis of flavonol aglycone profiles in T-DNA insertion lines.A**) UPLC traces of hydrolyzed extracts prepared from 5-day-old seedlings, with arrows indicating the retention times of the flavonols, quercetin (Q) and kaempferol (K). **B**) Comparison of kaempferol and quercetin levels determined from integrated peak areas.

Hydrolyzed flavonol extracts were analyzed by Ultra Performance Liquid Chromatography (UPLC) to provide phenotypic evidence of the gene disruptions identified by PCR. Five of the lines, *tt4-13, tt5-2, tt5-3, tt6-3* and *fls1-3,* had no detectable levels of kaempferol or quercetin, the two major flavonol aglycones found in *Arabidopsis* (Figure 4). All five alleles affect enzymes upstream of flavonol production in the flavonoid biosynthetic pathway. As in previous analyses of the *tt7-1* allele in the Landsberg (Ler) background, which lacks the F3^′^H enzyme, *tt7-5* in Col-0 also accumulated high levels of kaempferol but no detectable quercetin [46]. This is consistent with the catalytic role of F3^′^H in converting dihydrokaempferol to dihydroquercetin. The *tt11-11* and *ban-4* mutants contain insertions in the *ANS* and *ANR* genes, respectively. Both lines accumulated flavonols at levels comparable to wild type but displayed other phenotypes characteristic of defects in the respective genes. The *tt11-11* seeds exhibited an intermediate *tt* phenotype (Figure 1), but adult plants were devoid of red pigmentation, consistent with an absence of anthocyanins, while *ban-4* exhibited a red seed coat in immature seeds and a darker black seed coat in fully desiccated seeds, as described previously for *ban-1*[41].

## Conclusions

The flavonoid mutants described in this report represent a useful toolset for the study of many aspects of plant metabolism, cell biology, and physiology. The flavonoid pathway provides a unique model system for studying metabolic pathways as it has been well-characterized in a variety of model organisms and is essential for a wide range of cellular and physiological processes. Mutations for genes encoding many of the enzymes now exist in a uniform genetic background. While this communication focuses on flavonoid biosynthetic enzymes, mutant alleles exist for genes involved in mediating other aspects of flavonoid metabolism, including transcriptional regulation of gene expression and modification and cellular transport of pathway end products [47,48].

The flavonoid enzymes disrupted by T-DNA insertions have been hypothesized to participate in metabolic channeling via protein-protein interactions [7,49]. These mutations, all within the same genetic background, could greatly enhance our understanding of the regulation and dynamics of this channeling, which has broad reaching implications across metabolic research areas. The CHS mutant allele, *tt4-13,* has already been used by our group and others to further probe the involvement of this pathway in modulating the distribution of auxin and ethylene within *Arabidopsis* seedling roots [7,29,36,50], to characterize the distribution of flux among branch pathways of flavonoid metabolism [45], and to identify molecules that promote pollen fertilization in *Arabidopsis*[51]. The CHI allele, *tt5-3,* has been used in a metabolic profiling analysis of the response to UV light [52], whereas *tt5-2* was used to demonstrate a requirement for this CHI gene, among flavonoid genes, for flavonol synthesis in pollen [30]. The flavanone 3-hydroxylase mutant, *tt6-3,* has been used to characterize the biochemical activities of Arabidopsis F3H and Sorghum FNS [33,53], while the flavonoid 3^′^-hydroxylase line, *tt7-5,* was used by our group in the auxin-ethylene study [36], and *tt11-11,* was already used several years ago to show that *TDS4* is allelic to *tt18* (now renamed *tt11* per the findings of [38]) and encodes LDOX/ANS. The F3H and LDOX lines, *tt6-3* and *tt11-11,* have been used to demonstrate the utility of a novel metabolic profiling method for intact seed [54].

The collection of *tt* mutants presented here represent a means to several ends. As our understanding of the roles flavonoid compounds play in human health evolve, so too may our need to develop new crop lines to deliver increased amounts of these compounds in our diet. In addition, the flavonoid pigmentation compounds are of great horticultural importance. For these two reasons alone a thorough understanding of the dynamic metabolic processes involved in flavonoid production is important, but there are broader benefits to many areas of cellular and plant biology.

## Methods

### Analysis of flavonol profiles

*Arabidopsis* (Columbia ecotype) wild-type and transgenic seeds were surface-sterilized as described previously [25]. Approximately 5 mg of seeds were dispersed on agar plates containing Murashige and Skoog salts with 1% sucrose and incubated 2 d in the dark at 4°C. The seeds were then grown on the surface of the agar medium under continuous white light (100 μE m^-2^ s^-1^) at 21°C as previously described [55]. Flavonols were extracted from frozen tissue by grinding 20 seedlings in 200 μl 1% acetic acid in 80% methanol and incubating overnight at 4°C. The samples were clarified by centrifugation twice at 13,000 rpm, 4°C for 15 min each time. The samples were hydrolyzed as described in Burbulis et al. [21], followed by the addition of an equal volume of 100% methanol and centrifugation as before.

Flavonols in wild-type and transgenic seedlings were profiled using a Waters Acquity UPLC system with a UPLC phenyl C18 column (2.1 mm x 100 mm, Waters) and a linear elution gradient from 100% solvent A (0.1% formic acid in water) to 40% solvent B (0.1% formic acid in acetonitrile) over 13 min at 4°C, modified from Yonekura-Sakakibara [56]. Chromatograms were collected at 320 nm and 365 nm.

### Confirmation of knockouts by T-DNA insertion

Lines for each *tt* allele in Col-0 were ordered from the Arabidopsis Biological Resource Center (ABRC; The Ohio State University) and bred to homozygosity from a segregating population. The mapped locations of each T-DNA insertion were created using the T-DNA flanking sequence identified via the ABRC sequence viewer (Figure 2). To confirm that each line was homozygous, genomic DNA was extracted from one large leaf from each plant according to Edwards et al. [57] with slight modifications. Genomic DNA from Col-0 wild-type plants of approximately the same age was also extracted in the same manner to serve as a control template. Extracted genomic DNA was resuspended overnight in 100 μl ddH_2_O. PCR was performed using 1 to 2 μl of each sample with the primers listed in Table 2 in a total volume of 10–20 μl. PCR products were analyzed by agarose gel electrophoresis. Seeds for the *tt5-3, tt7-5,* and *tt11-11* homozygous lines have been deposited with the ABRC; homozygous lines are available at the ABRC for the other four lines through the SALK Confirmed T-DNA Project.

**Table 2:** Primers used for confirmation of homozygous lines

**Allele**	**Primer sequence** (5'-3^′^)	
CHS: *tt4-13*	Intact Gene	GATCACTCATGTCGTCTTCTG
(SALK_020583)		AGGGCCAGGCGGTGAAG
	T-DNA insertion	GATCACTCATGTCGTCTTCTG
		TTAGAGAGGAACGCTGTGC
CHI: *tt5-2*	Intact Gene	ATGTCTTCATCCAACGCCTG
(CS300857)		GTTCTCTTTGGCTAGTTTTTC
	T-DNA insertion	ATGTCTTCATCCAACGCCTG
CHI: *tt5-3*	Intact Gene	CGAAAGTAAGAATTAGAGAATAC
(SALK_034145)		AGGGCCAGGCGGTGAAG
	T-DNA insertion	CGAAAGTAAGAATTAGAGAATAC TGATAAACTTCTCAAACGCAC
F3H: *tt6-3*	Intact Gene	TGGTAGGTAGCTAGCGAC
(SALK_113904)		AACACACCGCGCCTAGC
	T-DNA insertion	TGGTAGGTAGCTAGCGAC
		AGGGCCAGGCGGTGAAG
F3^′^H: *tt7-5*	Intact Gene	CAGCGGATTGGAATTTGAAC
(SALK_053394)		CAGCTGTGAACATGTTCTG
	T-DNA insertion	GGACCGCTTGCTGCAACT
		CAGCTGTGAACATGTTCTG
ANS: *tt11-11*	Intact Gene	AGAGTTGAGAGTCTAGC
(SALK_073183)		GCAAAAGTCCGTGGAG
	T-DNA insertion	AGAGTTGAGAGTCTAGC
		TGGTTCACGTAGTGGGCCATCG
ANR: *ban-4*	Intact Gene	TGGACCAGACTCTTAC
(SALK_040250)		AGACCGGTCACATGC
	T-DNA insertion	AGACCGGTCACATGC
		TGGTTCACGTAGTGGGCCATCG

## Abbreviations

ANR: anthocyanidin reductase; ANS: anthocyanidin synthase; BAN: Banyuls; CHI: chalcone isomerase; CHS: chalcone synthase; F3^′^H: flavonoid 3^′^-hydroxylase; FLS: flavonol synthase; F3H: flavanone 3-hydroxylase; *tt*: *transparent testa*; UPLC: Ultra performance liquid chromatography.

## Competing interests

The authors declare that they have no competing interests.

## Authors’ contributions

PAB characterized several of the T-DNA insertions by PCR, produced the photograph showing the differences in seed coat color, and drafted the manuscript. MVR characterized several additional T-DNA insertions by PCR and participated in editing the manuscript. MM and RFH contributed the UPLC analysis. BSJW designed the study and participated in drafting and editing the manuscript. All authors read and approved the final manuscript.
